# Telomere biology disorder presenting acutely with pulmonary fibrosis and hepatopulmonary syndrome in a young adult male

**DOI:** 10.1002/rcr2.1182

**Published:** 2023-06-30

**Authors:** Samantha Chin‐Yun Kung, Olivia Dixon, Sarah Kentwell, Raja S. Vasireddy, Jonathan Rodgers, Yuming Ding, Tony Rahman, Caroline Tallis, Ian A. Yang, John A. Mackintosh

**Affiliations:** ^1^ Department of Thoracic Medicine The Prince Charles Hospital Brisbane Queensland Australia; ^2^ Department of Haematology The Children's Hospital at Westmead, The Sydney Children's Hospitals Network Sydney New South Wales Australia; ^3^ Genetic Health Queensland Royal Brisbane and Women's Hospital Brisbane Queensland Australia; ^4^ Faculty of Medicine The University of Queensland Brisbane Queensland Australia; ^5^ Gastroenterology and Hepatology The Prince Charles Hospital Brisbane Queensland Australia; ^6^ Department of Gastroenterology and Hepatology Princess Alexandra Hospital Brisbane Queensland Australia

**Keywords:** cirrhosis, hepatopulmonary syndrome, pulmonary fibrosis, telomeres

## Abstract

A 33‐year‐old man presented with acute dyspnoea and profound hypoxaemia, and had clubbing, greying of hair, orthodeoxia and fine inspiratory crackles. CT chest showed established pulmonary fibrosis in a usual interstitial pneumonia pattern. Additional investigations revealed a small patent foramen ovale, pancytopenia, and oesophageal varices and portal hypertensive gastropathy from liver cirrhosis. Telomere length testing demonstrated short telomeres (<1st percentile), confirming the diagnosis of a telomere biology disorder. An interstitial lung disease gene panel identified a pathogenic variant in *TERT* (c.1700C>T, p.(Thr567Met)) and a variant of uncertain significance in *PARN* (c.1159G>A, p.(Gly387Arg)). Combined lung and liver transplantation was deemed not suitable due to frailty and severe hepatopulmonary syndrome, and he died 56 days after presentation. Early recognition of the short telomere syndrome is important, and its multi‐organ involvement poses challenges to management. Genetic screening may be important in younger patients with pulmonary fibrosis or in unexplained liver cirrhosis.

## Graphical abstract

A 33‐year‐old man presented with acute dyspnoea and orthodeoxia. CT chest showed established pulmonary fibrosis in a usual interstitial pneumonia pattern and additional investigations revealed oesophageal varices and portal hypertensive gastropathy from liver cirrhosis. Telomere length testing demonstrated short telomeres (<1st percentile) with a gene panel identifying a pathogenic variant in TERT and a variant of uncertain significance in PARN.

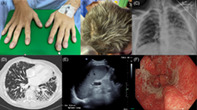

## INTRODUCTION

Familial pulmonary fibrosis is defined as the occurrence of any fibrotic interstitial lung disease (ILD) in at least two first or second‐degree family members.^1^ Mutations in genes involved in telomere maintenance are most frequently reported in this context. Owing to the presence of chromosomes and therefore telomeres in almost all cells in the human body, individuals and families with telomere biology disorders may manifest a variety of clinical manifestations in pulmonary and extra‐pulmonary tissues. Identification of a telomere biology disorder has important implications for the management of an affected individual and counselling of their family members. In this case report, we describe a young man and his family who manifested pulmonary and extra‐pulmonary features of a confirmed telomere biology disorder.

## CASE REPORT

**FIGURE 1:**
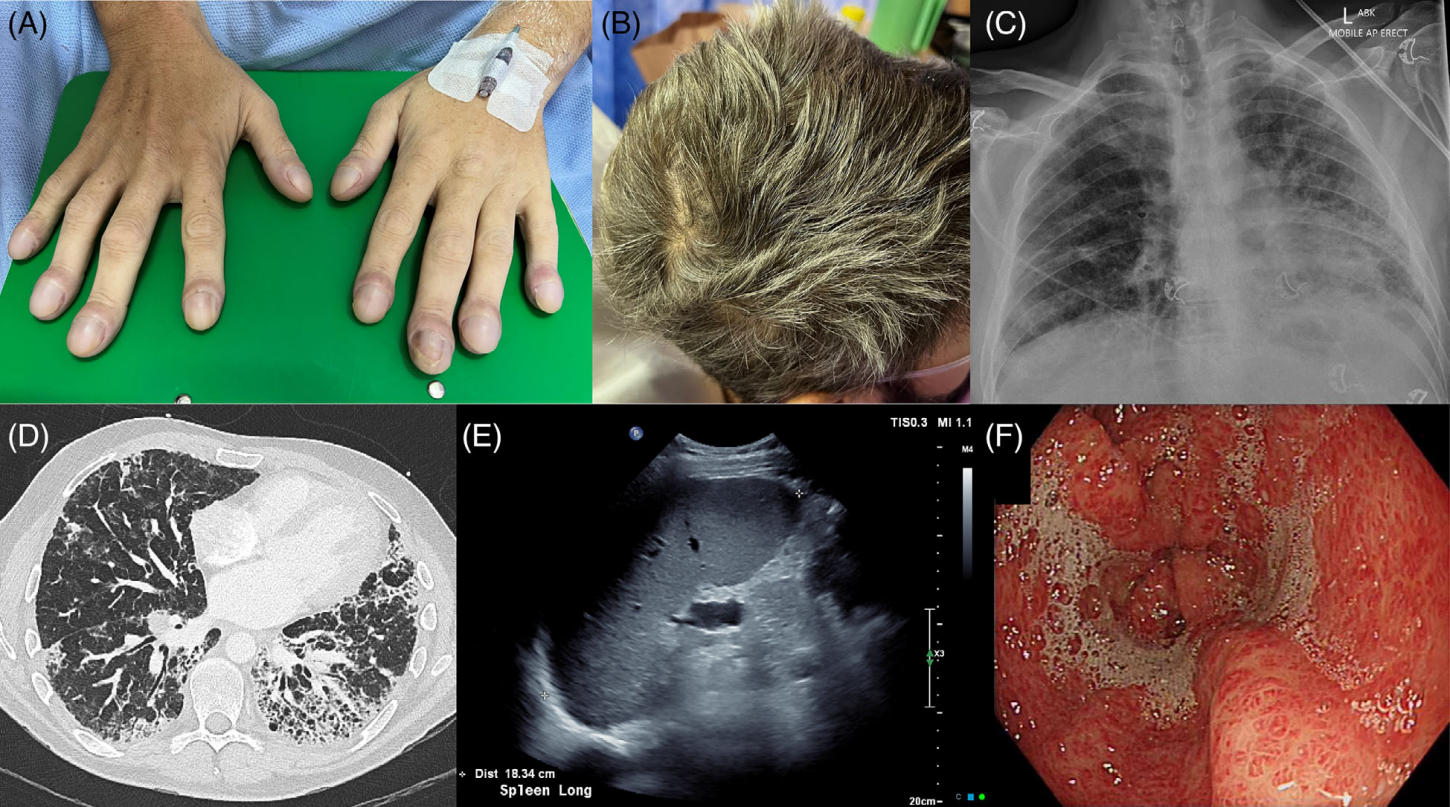
A 33‐year‐old man with pulmonary fibrosis and hepatopulmonary syndrome secondary to a telomere biology disorder. Clinical examination findings included (A) clubbing and (B) greying of hair, with (C) chest x‐ray demonstrating bilateral widespread patchy reticular opacities, more pronounced in the left lung. (D) High‐resolution computed tomography revealed a usual interstitial pneumonia pattern of pulmonary fibrosis. Cirrhosis with portal hypertension was confirmed by the presence of (E) splenomegaly on ultrasound and (F) portal hypertensive gastropathy identified during upper endoscopy.

A 33 year old male with no previous medical history presented with acute dyspnoea and profound hypoxaemia, having worked as a labourer up to the day prior to presentation. Despite this, he appeared comfortable at rest with oxygen supplementation via high‐flow nasal cannulae with a fraction of inspired oxygen of 0.93. Examination findings included clubbing, greying of hair, orthodeoxia and fine inspiratory crackles on chest auscultation (Figure 1).

In stark contrast to the acuity of his presentation, CT imaging of his chest demonstrated established pulmonary fibrosis in a usual interstitial pneumonia pattern (Figure 1). There were no changes to suggest an acute infection or exacerbation and there were no pulmonary emboli present. Apart from former smoking, there were no relevant exposures and a connective tissue disease screen was negative. Family history included his mother who had recently died of liver cirrhosis and a maternal uncle who died of interstitial lung disease and whose son had unexplained liver cirrhosis.

In the workup of orthodeoxia, a small patent foramen ovale was identified, but not deemed sufficient in size to explain his profound orthodeoxia. After an episode of melaena, an upper endoscopy identified grade one oesophageal varices and portal hypertensive gastropathy which revealed the presence of portal gastropathy from Child Pugh B liver cirrhosis. His orthodeoxia was subsequently attributed to hepatopulmonary syndrome. The presence of pancytopenia prompted a bone marrow aspirate and trephine which was normal.

**FIGURE 2:**
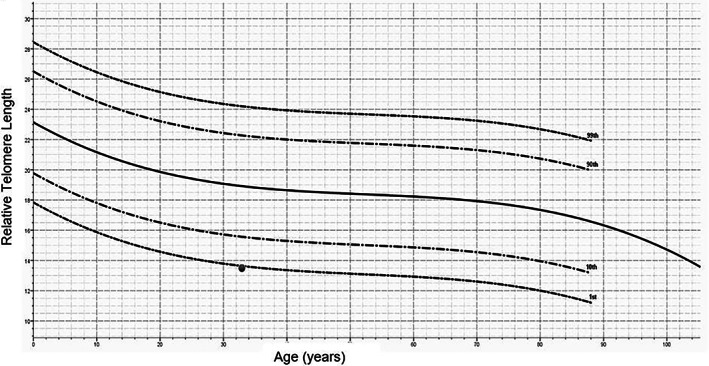
Relative telomere length (RTL) measured by Flow‐FISH method. Curved lines indicate the RTL of control cohort at 1st, 10th, 50th and 90th and 99th percentile. Coloured dot on the chart indicating the RTL of the individual tested in this case report.

Telomere length testing returned <1st percentile confirming the diagnosis of a telomere biology disorder (Figure 2). Genetic testing was performed prior to the patient's death using a clinical interstitial lung disease gene panel of 30 genes. This identified a pathogenic variant in *TERT* (c.1700C > T, p.(Thr567Met)) as well as a variant in *PARN* (c.1159G > A, p.(Gly387Arg)), classified as a variant of uncertain significance according to American College of Medical Genetics guidelines.

The patient underwent workup for combined lung and liver transplantation. He remained bedbound, owing to the orthodeoxia, and therefore was unable to partake in rehabilitation and rapidly became frail. He was ultimately deemed not suitable for combined organ transplantation due to frailty and severe hepatopulmonary syndrome. He was transferred to a palliative care unit where he died 56 days after initial presentation.

## DISCUSSION

This case highlights the complexities encountered when managing patients with telomere biology disorders where there is multi‐organ involvement. In this particular case, the presence of cirrhosis with hepatopulmonary syndrome was particularly problematic. Profound hypoxaemia with orthodeoxia and functional limitation were greater than would be expected due to pulmonary fibrosis alone, with hypoxaemia refractory to supplemental oxygen. Further compounding reduced tissue oxygen delivery was anaemia as a result of bleeding from portal hypertensive gastropathy, a known complication of portal hypertension. The ultimate result of this was poor functional capacity rapidly resulting in frailty, which was prohibitive to combined lung‐liver transplantation.

Hepatic impairment also carries implications for therapies available in the treatment of pulmonary fibrosis. Anti‐fibrotic agents undergo hepatic metabolism, thus there is potential for increased serum concentrations as well as drug‐induced liver injury^2^ in addition to pre‐existing cirrhosis.

Given the progressive nature and limited therapeutic options available for pulmonary fibrosis, lung transplantation is often pursued. However, this process is more complex in the setting of a telomere biology disorder. Multi‐organ transplantation (lung and liver) may be required, which can reduce the donor pool dramatically, leading to longer waitlist times. In this particular case, multiple blood transfusions were required due to gastrointestinal bleeding as a consequence of cirrhosis, which might contribute to HLA alloimmunisation.

Post‐transplantation, there may be an increased complication rate amongst patients with shortened telomeres. Acutely, there is potential for impaired wound healing and increased risk of immunosuppression‐related cytopenias, which may necessitate adjustments to immunosuppression.^3^, ^4^ In the longer term, there are increased risks of renal failure, malignancy and possibly shortened time to development of chronic lung allograft dysfunction, although this remains controversial.^3^, ^5^, ^6^

From a psychosocial standpoint, access to social support services and financial assistance is limited in younger patients, which places a significant burden on patients and their families. Despite the dismal prognosis without transplant and potential for rapid progression, the young age at presentation in patients with telomere biology disorders may be a barrier to appropriate discussions regarding prognosis and involvement of palliative care.

The European Respiratory Society (ERS) has recently published a statement on familial pulmonary fibrosis.^1^ Familial pulmonary fibrosis is defined as any fibrotic ILD occurring in at least two first or second‐degree family members and in this context, the authors of the ERS statement usually offer genetic sequencing. Additionally, the ERS statement authors offer genetic sequencing in any patient suspected of having a short telomere syndrome or where an idiopathic fibrotic ILD is diagnosed before the age of 50 years. The case described in this manuscript, therefore, meets multiple indications for genetic sequencing. In addition to the implications for a patient's own prognosis and management, a genetic diagnosis also allows predictive genetic testing to determine whether other family members are at risk and require their own anticipatory surveillance. The *TERT* variant has been reported in association with telomere biology disorders in the past. The *TERT* variant alone may account for the fulminant nature of our patient's features compared to his family due to anticipation, a phenomenon where each subsequent generation is affected earlier and with more severe features as they inherit progressively shorter telomeres from their parent. Alternatively, while there is little evidence to date of digenic inheritance in telomere biology disorders, it is possible that the *PARN* VUS may be pathogenic and exacerbate the clinical presentation. Further functional testing would be required to confirm this scenario which would significantly alter the genetic counselling provided to the family.

In summary, this case highlights the importance of early recognition of the short telomere syndrome and its complex multi‐system involvement posing challenges to management. While screening for shortened telomeres and associated mutations is not routine in either pulmonary fibrosis or cirrhosis, it is interesting to speculate on whether the identification of a telomerase gene mutation in the mother or maternal uncle may have enabled earlier diagnosis in this case. The discovery of a pathogenic telomerase gene mutation and variant of uncertain significance now have important implications for the other members of this family.

## AUTHOR CONTRIBUTIONS

All authors contributed equally to the drafting of this manuscript and approve its content.

## CONFLICT OF INTEREST STATEMENT

None declared.

## ETHICS STATEMENT

The authors declare that appropriate written informed consent was obtained for the publication of this manuscript and accompanying images.

## Data Availability

Data sharing not applicable to this article as no datasets were generated or analysed during the current study.
